# Converging Evidence From Electrocorticography and BOLD fMRI for a Sharp Functional Boundary in Superior Temporal Gyrus Related to Multisensory Speech Processing

**DOI:** 10.3389/fnhum.2018.00141

**Published:** 2018-04-24

**Authors:** Muge Ozker, Daniel Yoshor, Michael S. Beauchamp

**Affiliations:** ^1^Department of Neurosurgery, Baylor College of Medicine, Houston, TX, United States; ^2^Michael E. DeBakey Veterans Affairs Medical Center, Houston, TX, United States

**Keywords:** multisensory, speech perception, temporal lobe, electrocorticography (ECoG), BOLD fMRI, audiovisual speech perception, multisensory integration, speech in noise

## Abstract

Although humans can understand speech using the auditory modality alone, in noisy environments visual speech information from the talker’s mouth can rescue otherwise unintelligible auditory speech. To investigate the neural substrates of multisensory speech perception, we compared neural activity from the human superior temporal gyrus (STG) in two datasets. One dataset consisted of direct neural recordings (electrocorticography, ECoG) from surface electrodes implanted in epilepsy patients (this dataset has been previously published). The second dataset consisted of indirect measures of neural activity using blood oxygen level dependent functional magnetic resonance imaging (BOLD fMRI). Both ECoG and fMRI participants viewed the same clear and noisy audiovisual speech stimuli and performed the same speech recognition task. Both techniques demonstrated a sharp functional boundary in the STG, spatially coincident with an anatomical boundary defined by the posterior edge of Heschl’s gyrus. Cortex on the anterior side of the boundary responded more strongly to clear audiovisual speech than to noisy audiovisual speech while cortex on the posterior side of the boundary did not. For both ECoG and fMRI measurements, the transition between the functionally distinct regions happened within 10 mm of anterior-to-posterior distance along the STG. We relate this boundary to the multisensory neural code underlying speech perception and propose that it represents an important functional division within the human speech perception network.

## Introduction

The human ability to understand speech is one of our most important cognitive abilities. While speech can be understood using the auditory modality alone, vision provides important additional cues about speech. In particular, the mouth movements made by the talker can compensate for degraded or noisy auditory speech (Sumby and Pollack, 1954; Bernstein et al., 2004; Ross et al., 2007). While it has been known since Wernicke that posterior lateral temporal cortex is important for language comprehension, the advent of blood-oxygen level dependent functional magnetic resonance imaging (BOLD fMRI) led to important advances, such as the discovery that multiple regions in temporal cortex are selective for human voices (Belin et al., 2000). However, BOLD fMRI suffers from a major limitation, in that it is a slow and indirect measure of neural function. Spoken speech contains five or more syllables per second, requiring the neural processes that decode each syllable to be completed in less than 200 ms. In contrast, the sluggish hemodynamic response that underlies BOLD fMRI does not peak until several seconds after the neural activity that prompted it.

This drawback underscores the importance of complementing fMRI with other techniques that directly measure neural activity. The non-invasive techniques of EEG and MEG have led to a better understanding of the temporal dynamics of speech perception (Salmelin, 2007; Shahin et al., 2012; Crosse et al., 2016; Sohoglu and Davis, 2016). Recently, there has also been tremendous interest in electrocorticography (ECoG), a technique in which electrodes are implanted in the brains of patients with medically intractable epilepsy. Compared with EEG and MEG, ECoG allows localization of activity to the small population of neurons nearest each electrode, leading to the discovery of selective responses in the superior temporal gyrus (STG) for various speech features, including categorical representations of speech (Chang et al., 2010) phonetic features (Mesgarani et al., 2014) and prosody (Tang et al., 2017).

While the broad outlines of the organization of visual cortex are well-established (Grill-Spector and Malach, 2004), the layout of auditory cortex is less well known. Early areas of auditory cortex centered on Heschl’s gyrus contain maps of auditory frequency and spectral temporal modulation (Moerel et al., 2014). In contrast, within auditory association cortex in the STG, organization by auditory features is weaker, and the location and number of different functional areas is a matter of controversy (Leaver and Rauschecker, 2016). Recently, we used ECoG to document a double dissociation between anterior and posterior regions of the STG (Ozker et al., 2017). Both regions showed strong responses to audiovisual speech, but the anterior area responded more strongly to speech in which the auditory component was clear while the posterior area showed a different response pattern, responding similarly to clear and noisy speech or even responding more strongly to noisy audiovisual speech. There was a sharp anatomical boundary, defined by the posterior edge of Heschl’s gyrus, between the two areas. All electrodes anterior to the boundary responded more to clear speech and no electrodes posterior to the boundary did. These results were interpreted in the conceptual framework of multisensory integration. Auditory association areas in anterior STG respond strongly to clear auditory speech but show a reduced response because of the reduced information available in noisy auditory speech, paralleling the reduction in speech intelligibility. Multisensory areas in posterior STG are able use the visual speech information to compensate for the noisy auditory speech, restoring intelligibility. However, this demands recruitment of additional neuronal resources, leading to an increased response during noisy audiovisual speech perception.

While there have been numerous previous fMRI studies of noisy and clear audiovisual speech (e.g., Callan et al., 2003; Sekiyama et al., 2003; Bishop and Miller, 2009; Stevenson and James, 2009; Lee and Noppeney, 2011; McGettigan et al., 2012), none described a sharp boundary in the response patterns to clear and noisy speech within the STG. BOLD fMRI has the spatial resolution necessary to detect fine-scale cortical boundaries, such as between neighboring ocular dominance columns (Cheng et al., 2001), ruling out sensitivity of the technique itself as an explanation. Instead, we considered two other possibilities. One possible explanation is that the analysis or reporting strategies used in previous fMRI studies (such as group averaging or reporting only activation peaks) could have obscured a sharp functional boundary present in the fMRI data. A second, more worrisome, explanation is that the sharp boundary observed with ECoG reflects anomalous brain organization in the ECoG participants. Brain reorganization due to repeated seizures could have resulted in different STG functional properties in epileptic patients compared with healthy controls (Janszky et al., 2003; Kramer and Cash, 2012).

To distinguish these possibilities, in the present manuscript, we compare the ECoG dataset previously published in Ozker et al. (2017) with a new BOLD fMRI dataset not previously published. The healthy controls in the fMRI experiment viewed the same clear and noisy audiovisual speech stimuli viewed by the ECoG patients, and both set of participants performed the identical speech identification task. The BOLD fMRI data was analyzed without any spatial blurring or group averaging to ensure that these would not obscure areal boundaries within the STG. fMRI samples the entire brain volume, instead of the limited coverage obtained with ECoG electrodes, allowing for an examination of the responses to clear and noisy speech across the entire length of the STG.

## Materials and Methods

All participants (see Table 1 for demographic information) provided written informed consent and underwent experimental procedures approved by the Baylor College of Medicine (BCM) Institutional Review Board.

**Table 1:** Subject demographics.

Participant group	*N*	Age (mean)	Age (range)	Gender
ECoG	5	31	21–51	3F, 2M
fMRI	6	25	19–31	3F, 3M

**Figure 1:**
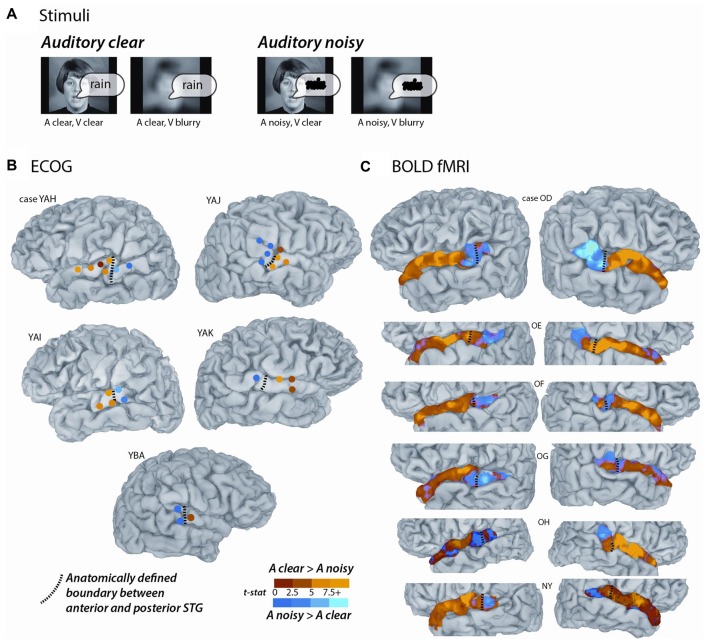
Converging evidence from functional magnetic resonance imaging (fMRI) and electrocorticography (ECoG) for a functional boundary in posterior superior temporal gyrus (STG). **(A)** The stimuli consisted of single-word recordings of audiovisual speech. In the four stimulus conditions, the auditory component was either clear (A clear) or replaced with speech-shaped noise (A noisy) and the visual component was either clear (V clear) or blurred (V blurry). The main analysis consisted of the contrast between the two A clear conditions and the two A noisy conditions. **(B)** Cortical surface models of five hemispheres from five ECoG participants (case letter codes indicate anonymized participant IDs). Colored circles show locations of subdural electrodes on the STG showing a significant response to audiovisual speech. Warm electrode colors indicate greater response to audiovisual speech with a clear auditory component. Cool electrode colors indicate greater response to speech with a noisy auditory component. Dashed black line shows the location of the anatomical border between anterior STG and posterior STG defined by the Desikan-Killiany atlas (Desikan et al., 2006). Adapted from Ozker et al. (2017). **(C)** Cortical surface models of 12 hemispheres from six fMRI participants. Surface nodes on the STG are colored by their preference for clear or noisy audiovisual speech (same color scale for **B,C**).

For the main experiment, identical stimuli were used for the ECoG and fMRI participants. The stimuli consisted of audiovisual recordings of a female talker from the Hoosier Audiovisual Multi-Talker Database speaking single words (“rain” or “rock”) in which the auditory component was either unaltered (auditory-clear) or replaced with speech-specific noise that matched the spectrotemporal power distribution of the original auditory speech (auditory-noisy). A parallel manipulation was performed on the visual component of the speech by replacing the original video with a blurred version. The two types of auditory speech and two types of visual speech were combined, resulting in four conditions (auditory-clear + visual-clear; auditory-clear + visual-blurred; auditory-noisy + visual-clear; auditory-noisy + visual-blurred). Schematic depictions of the stimuli are shown in Figure 1A, and the actual stimuli used in the experiments can be downloaded from https://openwetware.org/wiki/Beauchamp:Stimuli.

The face of the talker subtended approximately 15° of visual angle horizontally and vertically. For the ECoG participants, stimuli were viewed on a 15″ LCD monitor positioned at 57-cm distance from the participant and auditory stimuli were played through wall-mounted loudspeakers. For the fMRI participants, stimuli were viewed on a 32″ BOLDview LCD Screen (Cambridge Research Systems) located behind the scanner and viewed through a mirror affixed to the head coil (approximately 77-cm from screen to participant). Auditory stimuli were presented through stereo insert headphones (Sensimetrics). For both ECoG and fMRI participants, sound levels were adjusted to a comfortable volume before the experiment. The duration of each video clip was 1.4 s and the duration of the auditory stimulus was 520 ms for the “rain” stimulus and 580 ms for the “rock” stimulus. The auditory word onsets occurred after the video onset, 410 ms for “rain” and 450 ms for “rock”.

For the ECoG participants, from 32 to 56 repetitions of each condition were presented in random order. For the fMRI participants, 60 repetitions of each condition were presented in random order. Following each stimulus presentation, participants performed a two-alternative forced choice on the identity of the presented word.

### Definition of Anterior and Posterior Superior Temporal Gyrus (STG)

Cortical surface models were constructed from the high-resolution T1-weighted anatomical MRI scans of ECoG and fMRI participants using FreeSurfer (Fischl et al., 2001). For ECoG participants, the post-implantation computed tomography (CT) scan showing the electrode locations was registered to the anatomical MRI to ensure accurate electrode localization.

Two atlases were used to parcellate the STG. The Destrieux atlas defines the entire STG using the “G_temp_sup-Lateral” label (lateral aspect of the STG; Destrieux et al., 2010). The Desikan-Killiany atlas (Desikan et al., 2006) applies a single “Superior Temporal” label to both the STG and the STS with an additional “Banks Superior Temporal” label for the posterior portion of the STS, with an anterior border defined by the posterior-most point of Heschl’s gyrus. We cleaved the Destrieux STG into an anterior STG portion and a posterior STG portion using the Heschl’s gyrus landmark defined by the Desikan-Killiany atlas (boundary shown as black dashed lines in Figure 1). The posterior STG is continuous with the supramarginal gyrus. Since the two atlases vary in their handling of this boundary, we manually defined the posterior boundary of the posterior STG as being just past the location where the gyrus begins its sharp turn upward into parietal lobe. All analyses were done only within single participants without any normalization or spatial blurring. In order to report the location of the anterior-posterior boundary in standard space, individual MRIs were aligned to the N27 brain (Holmes et al., 1998).

### ECoG Experimental Design and Data Analysis

The ECoG dataset was previously published in Ozker et al. (2017). Experiments were conducted in the epilepsy monitoring unit of Baylor St. Luke’s Medical Center. Patients rested comfortably in their hospital beds while viewing stimuli presented on an LCD monitor mounted on a table and positioned at 57 cm distance from the participant. While the participants viewed stimulus movies, a 128-channel Cerebus amplifier (Blackrock Microsystems, Salt Lake City, UT, USA) recorded from subdural electrodes that consisted of platinum alloy discs (diameter 2.3 mm) embedded in a flexible silicon sheet with inter-electrode distance of 10 mm. An inactive intracranial electrode implanted facing the skull was used as a reference for recording. Signals were amplified, filtered and digitized at 2 kHz. Offline, common average referencing was used to remove artifacts, and the data was epoched according to stimulus timing. Line noise was removed and spectral decomposition was performed using multitapers. The measure of neural activity was the broad-band high-gamma response (70–110 Hz) measured as the percent change relative to a pre-stimulus baseline window (500–100 ms before auditory stimulus onset). The high-gamma broadband response was used as it is the ECoG signal most closely associated with the rate of action potentials and the BOLD fMRI response (reviewed in Ray and Maunsell, 2011; Lachaux et al., 2012; Ojemann et al., 2013). Across patients, a total of 527 intracranial electrodes were recorded from. Of these, 55 were located on the STG. Twenty-seven of these showed a minimal level of stimulus-related activity, defined as significant high-gamma responses to audiovisual speech compared with prestimulus baseline (*p* < 10^−3^, equivalent to 40% increase in stimulus power from baseline) and were included in the analysis.

### fMRI Experimental Design and Data Analysis

Experiments were conducted in the Core for Advanced MRI (CAMRI) at BCM using a 3 Tesla Siemens Trio MR scanner equipped with a 32-channel head gradient coil. BOLD fMRI data was collected using a multislice echo planar imaging sequence (Setsompop et al., 2012) with TR = 1500 ms, TE = 30 ms, flip angle = 72°, in-plane resolution of 2 × 2 mm, 69 2-mm axial slices, multiband factor: 3, GRAPPA factor: 2. fMRI data was analyzed using the afni_proc.py pipeline (Cox, 1996). Data was time shifted to account for different acquisition times for different slices; aligned to the first functional volume which was in turn aligned with the high-resolution anatomical; and rescaled so that each voxel had a mean of 100. No blurring or spatial normalization of any sort was applied to the EPI data. Five runs (scan series) were collected, each with 160 brain volumes (4 min duration). Each run contained 48 3-s trials, 12 for each stimulus condition, for a total of sixty repetitions of each condition. A rapid event-related design was used with fixation baseline occupying the remaining 96 s of each run, optimized with the scheduling algorithm optseq2^1^ (Dale et al., 1999).

**Figure 2:**
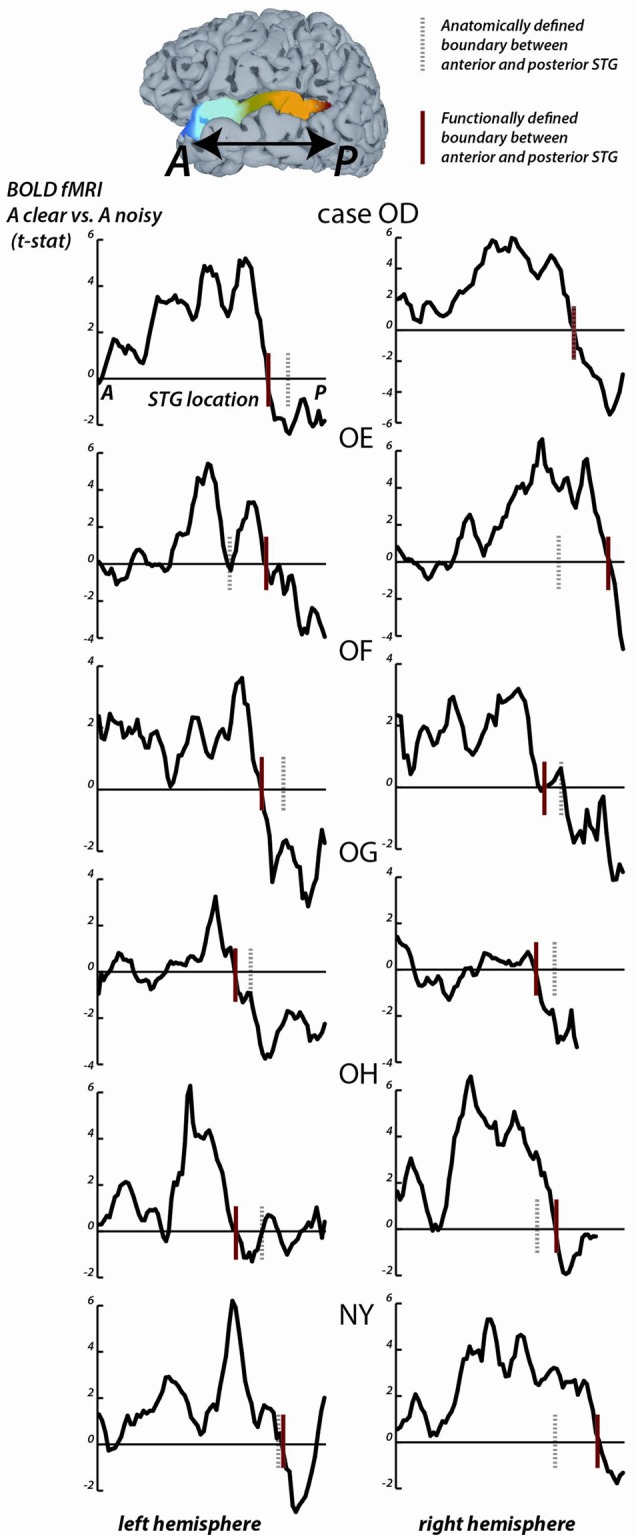
fMRI responses along the length of the STG. For each fMRI participant, the STG was parcellated into 1 mm bins from the most anterior point (A) to the most posterior point (P). For all surface nodes in each bin, the average value of the clear vs. noisy *t*-statistic was averaged. In each plot, the y-axis is the average *t*-statistic and the x-axis is the location along the STG from A to P. The left column shows plots for the left hemispheres, the right column shows plots for the right hemispheres; case letter codes indicate anonymized participant IDs. In each hemisphere, the location of the functional boundary between anterior STG and posterior STG was defined as the first zero-crossing of this curve in the posterior third of the STG (red vertical lines). In each hemisphere, the anatomical boundary between anterior and posterior STG was defined by the posterior margin of Heschl’s gyrus (gray dashed vertical lines). For case OD right hemisphere, the two boundaries overlap.

A generalized linear model was used to model the fMRI time series independently for each voxel using the 3dDeconvolve function in AFNI. The model contained 10 regressors: six regressors of no interest generated by the motion correction process and four regressors of interest (one for each stimulus condition) using an exponential hemodynamic response function (HRF) generated with the 3dDeconvolve option “BLOCK(2,1)”. A general linear test with the values of “+1 +1 −1 −1” was used to find the *t*-statistic for the contrast between the two conditions with clear auditory speech and the two conditions with noisy auditory speech (data in Figures 1, 2). This contrast between auditory-clear and auditory-noisy was the main dependent measure in the analysis.

For the STG length analysis (Figure 2), unthresholded fMRI data in the form of the clear vs. noisy *t-*statistic was mapped to the cortical surface using the AFNI function 3dVol2Surf. The options “-ave -f_steps 15” were used, resulting in a line between each node on the pial surface and the corresponding node on the smoothed white matter surface being subdivided into 15 equal segments, with the fMRI voxel values at each segment sampled and averaged. The entire STG was divided into 1 mm bins, from anterior to posterior, and the *t*-statistic at all nodes within each bin was averaged. For each hemisphere, a functional boundary was defined as the bin containing the first zero-crossing of the *t-*statistic (moving in an anterior-to-posterior direction) in the posterior third of the STG.

To estimate the shape of the HRF without assumptions, a second model was constructed that used tent functions to estimate the amplitude of the response independently at each time point of the BOLD response. For the main fMRI experiment, the response window spanned 0–15 s after stimulus onset (11 time points at a TR of 1.5 s) using the 3dDeconvolve option “TENTzero(0,15,11)”, resulting in a model with 50 regressors (6 motion regressors and 44 regressors of interest).

To estimate the average BOLD fMRI HRF, anterior and posterior STG ROIs were created in each participant using as a boundary either the anatomical Heschl’s gyrus boundary or the functional boundary defined by the STG length analysis. The anterior STG ROI contained all voxels from 0 mm to 30 mm anterior to the boundary and the posterior STG ROI contained all voxels from 0 mm to 15 mm posterior to the boundary. These values were chosen for consistency with the ECoG electrode locations, which ranged from 30 mm anterior to the anatomical/functional boundary to 15 mm posterior to it (Figure 1B). For correspondence with the ECoG electrode selection criteria (in which only electrodes that showed some response were included in the analysis) only voxels with an omnibus Full-F statistic of *F* > 5 (*q* < 10^−6^) were included in the ROIs.

To directly compare the BOLD fMRI with the ECoG responses, the ECoG response were convolved with a double-gamma HRF with peak time = 6 s, undershoot time = 10 s and response-to-undershoot ratio = 4 (Lindquist et al., 2009). The only free parameter was a scale parameter that matched the amplitude of the predicted and actual fMRI responses; scale parameters that minimized the difference between the predicted and actual fMRI responses for each of the four curves were found using the Matlab function *fminbnd*.

### Linear Mixed-Effects Models

Linear mixed-effect models (LMEs) were constructed using *R* with the lme4 package. LMEs are similar to repeated-measure analysis of variances (ANOVAs) but have several advantages: LMEs are more statistically conservative, LMEs better handle missing observations and LMEs account for the correlation structure of the variables. The dependent measure for each LME was the % signal change from baseline. For the ECoG data, each electrode constituted an independent sample, and the responses to each stimulus condition were entered into the LME. For the fMRI data, each hemisphere constituted an independent sample, and the responses to each stimulus condition in the anterior and posterior STG ROIs in that hemisphere were entered into the LME. The fixed factors were location (anterior vs. posterior STG), the presence or absence of auditory noise (auditory-clear vs. auditory-noisy) and the presence or absence of visual blur (visual-clear vs. visual-blurry). For each fixed factor, the LME estimated the significance of the effect and the magnitude of the effect relative to a baseline condition, which was always the response to auditory-clear, visual-clear speech in anterior STG.

### Additional fMRI Data

Additional fMRI data was collected while participants were passively presented with 20-s excerpts from short stories (Aesop’s fables) presented in auditory-only, visual-only and audiovisual versions, all recorded by the same female talker (Nath et al., 2011; Nath and Beauchamp, 2012). Data with these stimuli were collected in two runs, each with 180 brain volumes (4:30 duration); for one participant, only one run was collected. Each run contained nine blocks (20 s of stimulus, 10 s of fixation baseline) consisting of three blocks each (in random order) of the three types of stimuli. The HRF was estimated using a response window spanning 0–30 s after stimulus onset (21 time points) using “TENTzero(0,30,21)” for each of the three stimulus types. The beta-weights at the 4.5 s, 6 s and 7.5 s time points were averaged to create the estimate of response amplitude.

## Results

Participants were presented with audiovisual speech stimuli with a clear or noisy auditory component and a clear or blurry visual component (Figure 1A). In the ECoG dataset, electrodes implanted in different locations on the STG responded differently. Electrodes on the anterior STG responded more strongly to audiovisual speech with a clear auditory component while electrodes on posterior STG did not (Figure 1B). The posterior-most point of Heschl’s gyrus has been proposed as a boundary dividing the STG/STS into anterior and posterior sections (Desikan et al., 2006; Ozker et al., 2017). All electrodes anterior to the boundary responded more strongly to clear speech while none of the electrodes posterior to the boundary did. The difference in response patterns was striking, even between electrodes that were only 10 mm apart, the closest possible distance in our recording array. For instance, in one participant the response to clear speech of an anterior electrode was more than double its response to noisy speech (138% ± 13% vs. 49% ± 5%, mean across trials ± SEM; unpaired *t*-test: *t*_109_ = +6.2, *p* = 10^−8^) while the adjacent electrode, located 10 mm posterior across the boundary, responded less than half as much to clear speech as noisy speech (38% ± 5% vs. 89% ± 9%, *t*_109_ = −4.5, *p* = 10^−5^). These effects were quantified across electrodes using a LME model (Table 2). There were three significant effects in the model: a main effect of location, driven by smaller responses in the posterior STG (*p* = 0.01, effect magnitude of 101%); a main effect of auditory noise, driven by weaker responses to noisy stimuli (*p* = 10^−13^, magnitude 110%); and an interaction between location and auditory noise, driven by a larger response to noisy auditory stimuli in posterior STG (*p* = 10^−10^, magnitude 141%).

**Table 2:** Linear mixed-effects (LMEs) model of the response amplitude in anterior and posterior electrocorticography (ECoG) electrodes.

Fixed effects	Estimate	Std. Error	DF	*t*-value	*p*-value
Baseline	183.1	24.8	33.7	*7.4*	*10*^−8^
**Auditory noise (An)**	**−109.6**	**13.5**	**188**	**−8.1**	**10^−13^**
**Posterior location × An**	**140.6**	**21.2**	**188**	**6.6**	**10^−10^**
**Posterior location**	**−10.1**	**38.7**	**34.2**	**−2.6**	**0.01**
Visual blur (Vb)	21.6	13.5	188	1.6	0.11
An × Vb	−13.3	19.1	188	−0.7	0.49
Posterior location × Vb	−8.9	21.2	188	−0.4	0.67
Posterior location × An × Vb	3.6	29.9	188	0.1	0.91

*Results of an LME model of the ECoG response amplitudes, reprinted from Ozker et al. (2017). The fixed effects were the location of each electrode (Anterior *vs*. Posterior), the presence or absence of auditory noise (An) in the stimulus and the presence or absence of visual blur (Vb) in the stimulus. Electrodes and stimulus exemplar were included in the model as random factors. For each effect, the model estimates (in units of percent signal change) for that factor are shown relative to baseline, the response in anterior electrodes to clear audiovisual speech (AV stimulus condition). The baseline is shown first; all other effects are ranked by absolute t value. Significant effects are shown in bold. The significance of the baseline fixed effect is italicized because it was prespecified: only electrodes with significant amplitudes were included in the analysis*.

To determine if a similar anterior-posterior STG boundary could be observed with fMRI, we scanned participants viewing the same stimuli as the ECoG participants and mapped the unthresholded statistical contrast of clear vs. noisy speech along the STG of each hemisphere (Figure 1C). Anterior STG responded more strongly to clear speech while posterior STG did not.

To quantify the location of the anterior-posterior boundary, the preference for clear vs. noisy audiovisual speech in unthresholded fMRI data was plotted in 1 mm bins along the entire anterior-to-posterior extent of the STG (Figure 2). The sign change in the *t-*statistic of the clear vs. noisy contrast was used to define a *functional* A-P boundary in the STG (red lines in Figure 2) for comparison with the* anatomical* A-P boundary in the STG defined by the posterior margin of Heschl’s gyrus (black lines in Figure 2).

The mean anterior-to-posterior location of the fMRI-defined functional boundary in standard space was *y* = −28 mm (± 9 mm SD). The mean standard space location of the atlas-defined anatomical boundary in these participants was *y* = −30 mm (±5 mm SD).

In some cases, the boundaries aligned remarkably well (inter-boundary distance of 1 mm, case OD right hemisphere) while in others they were farther apart (distance of 20 mm, case OE right hemisphere). There was no consistent anterior-to-posterior difference between the anatomical and functional boundaries, resulting in a small mean distance between them (*y* = −28 vs. −30, *t*_11_ = 0.2, *p* = 0.8).

The location of the anatomical and functional boundaries in the fMRI participants were similar to that of the anatomical boundary in the ECoG participants, located at *y* = −27 mm (±2 mm SD); the 1 cm spacing of the ECoG electrodes did not allow a separate estimate of the functional boundary.

As in the ECoG data, the fMRI transition between clear and noisy speech preferring cortex happened over a short cortical distance. For instance, in participant OD’s left hemisphere, the *t-*statistic of the clear vs. noisy contrast changed from *t* = +5 to *t* = −2 within 10 mm.

**Figure 3:**
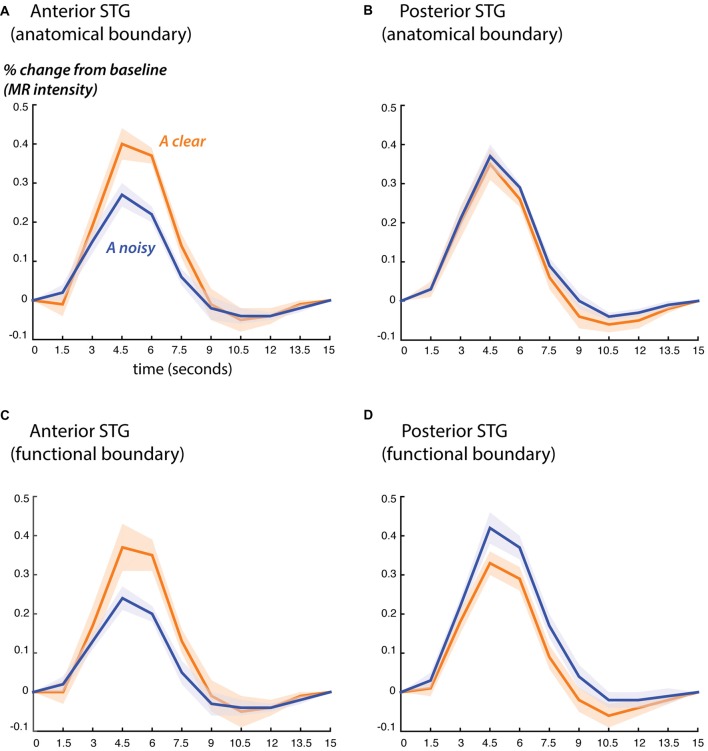
Blood oxygen level dependent (BOLD) fMRI responses to clear and noisy audiovisual speech in anterior and posterior STG. **(A)** The average fMRI response for anterior STG was created by selecting all voxels in each hemisphere that were located from 0 mm to 30 mm anterior to the anatomical boundary defined by Heschl’s gyrus and that showed a significant response to any stimulus. Responses shown for audiovisual speech with a clear auditory component (blue) and a noisy auditory component (orange). Lines show mean, shaded regions show SEM across participants. **(B)** The average fMRI response for posterior STG was created by selecting all responsive voxels in each hemisphere that were located from 0 mm to 15 mm posterior to the anatomical boundary defined by Heschl’s gyrus. **(C)** Average BOLD fMRI response in the anterior STG, defined as all responsive voxels located from 0 mm to 30 mm anterior to the functional boundaries defined as shown in Figure 2. **(D)** BOLD fMRI response in the posterior STG, defined as all responsive voxels located from 0 mm to 15 mm posterior to the functional boundary.

Next, we examined the fMRI response profiles on either side of the anatomical and functional boundaries (Figure 3). We classified the STG from 0 mm to 30 mm anterior to each boundary as “anterior” and the STG from 0 mm to 15 mm posterior to each boundary as “posterior”. These values were chosen for consistency with the ECoG electrode locations, which ranged from 30 mm anterior to the boundary to 15 mm posterior to it.

Both the anatomical and functionally-defined STG ROIs showed the characteristic BOLD response, with a positive peak between 4 s and 6 s and a negative post-undershoot at the 10.5 s time point. The responses were robust, with a peak between 0.2% and 0.4% for a single audiovisual word. While the anterior STG responded more strongly to clear speech, the posterior STG did not. Table 3 shows the results of the LME on the fMRI response amplitudes using an anatomically-defined border between anterior and posterior STG. The same three significant effects were observed as in the ECoG LME: a main effect of ROI location driven by smaller responses in the posterior STG (*p* = 0.01, magnitude 0.07%); a main effect of auditory noise driven by weaker responses to noisy auditory stimuli (*p* = 10^−6^, magnitude 0.14%); and an interaction between these factors driven by larger responses to noisy auditory stimuli in posterior STG (*p* = 10^−4^, magnitude 0.16%). The same effects were observed in an LME using the functionally-defined border between anterior and posterior STG (Table 4) with a significant main effect of auditory noise and interaction between auditory noise and location. However, the functionally-defined LME should be interpreted with caution as the border definition incorporated fMRI data, potentially biasing the model.

**Table 3:** LME model of the fMRI response amplitude in superior temporal gyrus (STG) regions defined by anatomical boundary.

Fixed effects	Estimate	Std. Error	DF	*t*-value	*p*-value
Baseline	0.40	0.03	35.9	*15.4*	<*10*^−16^
**Auditory noise (An)**	**−0.14**	**0.03**	**84**	**−5.4**	**10^−6^**
**Posterior location × An**	**0.16**	**0.04**	**84**	**4.2**	**10^−4^**
**Posterior location**	**−0.07**	**0.03**	**84**	**−2.6**	**0.01**
Visual blur (Vb)	−0.03	0.03	84	−1.0	0.31
Posterior location × Vb	−0.02	0.04	84	−0.6	0.52
Posterior location × An × Vb	0.01	0.05	84	0.2	0.83
An × Vb	0.00	0.04	84	0.0	0.97

*Results of an LME model of the fMRI response amplitude in STG regions defined by an anatomical boundary. The fixed effects were the location of each region (Anterior *vs*. Posterior), the presence or absence of auditory noise (An) in the stimulus and the presence or absence of visual blur (Vb) in the stimulus. STG ROIs from right and left hemisphere across six subjects were included in the model as random factor. For each effect, the model estimate (in units of % signal change) for that factor is shown relative to baseline, the response in anterior STG ROI to clear audiovisual speech. The baseline is shown first, all other effects are ranked by absolute t-value. Significant effects are shown in bold. The significance of the baseline fixed effect is italicized because it was prespecified*.

**Table 4:** LME model of the fMRI response amplitude in STG regions defined by functional boundary.

Fixed effects	Estimate	Std. Error	DF	*t*-value	*p*-value
Baseline	0.37	0.03	38.32	*10.8*	*10*^−13^
**Posterior location × An**	**0.21**	**0.05**	**84**	**4.1**	**10^−4^**
**Auditory noise (An)**	**−0.14**	**0.04**	**84**	**−3.8**	**10^−4^**
Posterior location	−0.04	0.04	84	−1.1	0.28
Visual blur (Vb)	−0.02	0.04	84	−0.7	0.49
Posterior location × Vb	−0.02	0.05	84	−0.4	0.66
Posterior location × An × Vb	0.03	0.07	84	0.4	0.69
An × Vb	−0.01	0.05	84	−0.1	0.92

*Results of an LME model of the fMRI response amplitude in STG regions defined by the functionally-defined boundary between anterior and posterior STG. Significant effects are shown in bold. The significance of the baseline fixed effect is italicized because it was prespecified*.

### Behavioral Data

Participants’ ability to identify the word presented in each trial was at ceiling for auditory-clear stimuli (mean 99%) and lower for auditory-noisy stimuli (mean 78%). This performance difference could drive differences in brain responses, if, for instance, error monitoring circuits were more engaged during poorer performance. To address this concern, we compared responses across stimulus conditions with identical auditory stimuli but differing performance. Accuracy for noisy auditory speech was better when paired with clear visual speech than when paired with blurry visual speech (87% vs. 69%, *p* = 0.004). A performance explanation predicts differing brain responses to these two types of stimuli (due to differing performance) while an auditory stimulus explanation predicts no difference (since the two stimuli have identical auditory components of the stimuli). LMEs on the brain responses did not show a significant difference between these conditions (*p* = 0.11 for ECoG, *p* = 0.31 for fMRI) suggesting that performance differences do not account for the observed differences in brain responses to clear and noisy auditory speech.

### Comparison Between fMRI and ECoG

**Figure 4:**
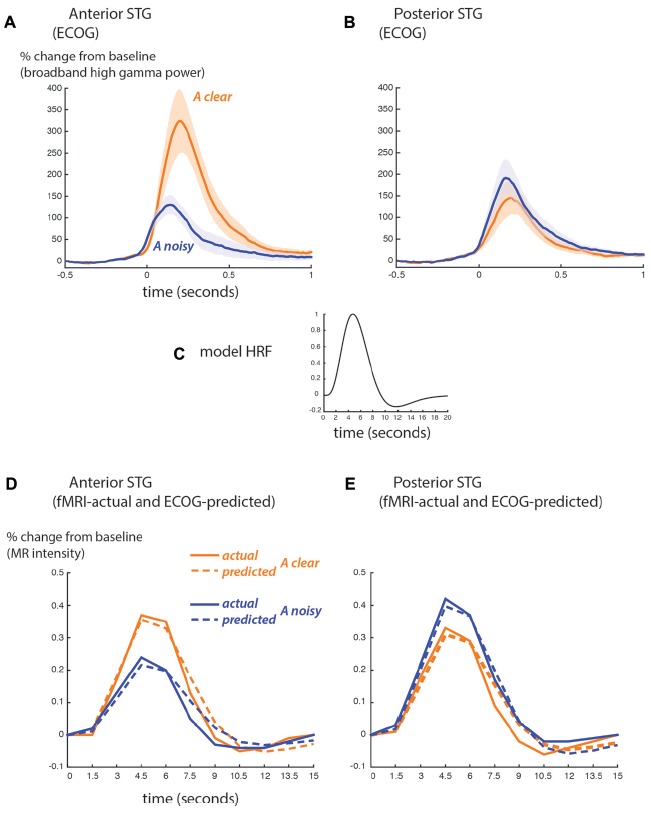
Comparison of ECoG and BOLD fMRI responses. **(A)** The broadband high-gamma power (70–110 Hz) in the ECoG response plotted as the increase relative to prestimulus baseline (−500 to −100 ms) for audiovisual speech with a clear auditory component (blue line) and a noisy auditory component (orange line). Grand mean across all anterior STG electrodes in all participants (shaded region shows SEM). Adapted from Ozker et al. (2017). **(B)** Grand mean response to clear and noisy speech across all posterior STG electrodes. **(C)** A model hemodynamic response function (HRF) used to create the shape of the predicted fMRI response by convolving with the ECoG response. **(D)** Predicted fMRI responses for anterior STG (dotted lines) compared with the actual fMRI responses from Figure 3C (solid lines). The predicted response was created by convolution with the model HRF and fitting a scale factor to determine the amplitude. A separate scale factor was used for each condition. **(E)** Predicted fMRI responses for posterior STG (dotted lines) compared against the actual fMRI responses from Figure 3D (solid lines).

BOLD fMRI and ECoG provide different measures of neural activity. BOLD fMRI is an indirect measure, with a time scale of seconds and an amplitude scale of image brightness increases relative to baseline. ECoG is a direct measure of neural activity, with a time scale of milliseconds and an amplitude scale of spectral power increases relative to baseline. To compare the fMRI and ECoG responses, we accounted for these differences, beginning with the time scale. Figure 4 shows the ECoG responses from the STG, reprinted from Figure 4 of Ozker et al. (2017). Increases in the high-gamma broadband signal began less than 100 ms after auditory speech onset, and peaked at about 200 ms after auditory speech onset. To convert the directly-recorded neural activity measured with ECoG to the indirect and much slower measure of neural activity provided by BOLD fMRI, the ECoG responses were convolved with a standard HRF (Figure 4C) and downsampled from 1 ms resolution to a temporal resolution of 1.5 s, the repetition time (TR) of the fMRI data. This created a predicted fMRI response (based on the measured ECoG responses) on the same time scale and time base as the actual fMRI response. The second obstacle was the different amplitude scales of the responses. ECoG amplitude is measured in % change in the high-gamma broadband signal relative to pre-stimulus fixation baseline, while fMRI signal amplitude is measured in % intensity increase of the EPI images relative to fixation baseline. A separate scale factor was calculated for each condition in order to generate the best fit between the predicted and actual fMRI responses.

Figure 4D shows the predicted-from-ECoG responses and actual fMRI responses based on the functional boundary between anterior and posterior STG. The shape of the responses was similar, as demonstrated by a high correlation coefficient between predicted and actual responses (anterior STG: 0.98 for auditory-clear, 0.96 for auditory-noisy; posterior STG: 0.97 for auditory-clear, 0.99 for auditory-noisy). The average across scale factor across conditions for the amplitude conversion was 612 ECoG% per BOLD%, meaning that a peak ECoG response of 612% was equivalent to a BOLD fMRI response of 1%. The scale factors were identical for auditory-clear and auditory-noisy conditions in posterior STG (476) but were markedly higher in anterior STG, especially for auditory-clear audiovisual words (909 for auditory-clear and 588 for auditory-noisy). This reflected the fact that in the ECoG data, the anterior STG response to clear speech was more than twice as large as the response to auditory-noisy speech (300% vs. 110%) while in fMRI, the anterior STG responded more strongly to clear speech than to noisy speech but the difference was less pronounced (0.37% vs. 0.24%).

**Figure 5:**
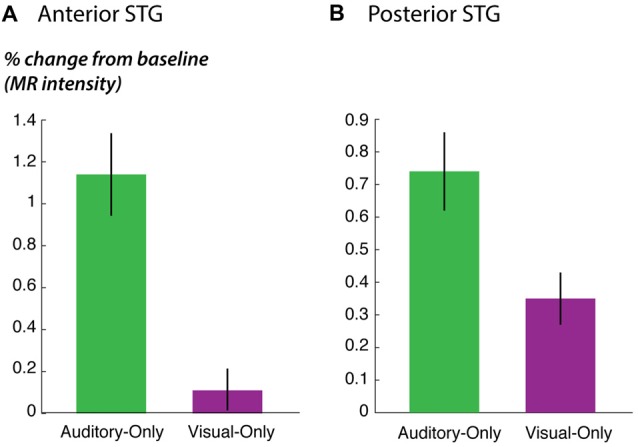
STG responses to unisensory auditory and visual speech. **(A)** The response of anterior STG to unisensory auditory and visual speech in the fMRI localizer experiment. **(B)** The response of posterior STG to unisensory auditory and visual speech in the fMRI localizer experiment.

In ECoG and fMRI, we observed distinct patterns of responses to clear and noisy speech in anterior and posterior STG. A possible explanation for these results is that anterior STG is unisensory auditory cortex, rendering it susceptible to auditory noise added to speech, while posterior STG is multisensory auditory-visual cortex, allowing it to compensate for auditory noise using visual speech information. This explanation predicts that posterior STG should show stronger responses to visual speech than anterior STG. To test this explanation, we took advantage of the fact that the fMRI participants viewed additional stimuli consisting of short stories presented in unisensory visual, unisensory auditory and audiovisual versions. The response to these stories was calculated in STG ROIs defined using the functional boundary (Figure 5). This analysis was unbiased because the functional boundary was created using fMRI data from auditory-clear and auditory-noisy words, completely independent of the story stimuli.

As predicted, the response to the unisensory visual story stimuli was significantly stronger in posterior STG than in anterior STG (posterior vs. anterior: 0.4% vs. 0.1%, *p* = 0.02 for visual speech). This could not be explained by an overall difference in responsiveness; in fact, for the other story stimuli, there was a trend towards weaker responses in posterior STG (posterior vs. anterior: 0.7% vs. 1.1%, *p* = 0.09 for unisensory auditory speech; 1.3% vs. 1.7%; *p* = 0.07 for audiovisual speech), consistent with the weaker responses in posterior STG to single audiovisual words observed in the main experiment (effect of posterior location in Table 3).

## Discussion

We measured neural activity in the human STG using two very different techniques: directly, using surface electrodes implanted in ECoG participants with epilepsy, or indirectly, using the BOLD response in fMRI participants who were healthy controls. Both ECoG and fMRI participants viewed the same clear and noisy audiovisual speech stimuli and performed the same speech recognition task. Both techniques demonstrated a sharp functional boundary in the STG. Cortex responded more strongly to clear audiovisual speech on the anterior side of the boundary while on the posterior side of the boundary it did not. For both techniques, the boundary was located at a similar location in standard space (*y* = 30 mm) and the transition between the two functional zones happened within 10 mm of anterior-to-posterior distance along the STG.

In both fMRI and ECoG patients, an anatomical boundary set at the most posterior point of Heschl’s gyrus provided a reasonable proxy for the functional boundary. This is important because unlike the fMRI or ECoG data needed to locate the functional boundary, the structural MRI scan needed to locate the anatomical boundary is easily obtainable (for instance, in the examination of patients with brain lesions). While primary visual and auditory cortex are easily localizable using anatomical landmarks, it has proven to be much more of a challenge to find landmarks for association areas (Weiner and Grill-Spector, 2011; Witthoft et al., 2014).

Multisensory integration provides a conceptual framework for understanding these results. When noisy auditory speech is presented, auditory information alone is insufficient for perception, and auditory-speech regions in anterior STG respond with diminished intensity. Visual speech information can compensate for noisy auditory speech (Sumby and Pollack, 1954; Bernstein et al., 2004; Ross et al., 2007), but this requires recruitment of multisensory brain regions capable of combining the auditory and visual speech information to restore intelligibility. While both anterior and posterior STG responded to audiovisual speech, data from the fMRI localizer experiment showed that posterior STG responded more strongly to visual-only speech than anterior STG, supporting the idea that posterior STG is a multisensory area capable of combining auditory and visual speech.

The neural code in posterior STG is hinted at by a recent study, which found that a region of posterior STG and STS (similar to the posterior STG region described in the present manuscript) responded more strongly to silent videos of faces making mouth movements compared to silent videos of faces making eye movements (Zhu and Beauchamp, 2017). The same region responded strongly to unisensory auditory speech, with a greater amplitude for vocal than non-vocal sounds. Interestingly, as statistical thresholds were increased to select voxels with a greater preference for visual mouth movements, response to unisensory auditory speech increased, suggesting that at a single voxel level, small populations of neurons code for mouth movements and speech sounds, the two components of audiovisual speech (Bernstein et al., 2011). This cross-modal correspondence in neural coding of multisensory cues is exactly as predicted by computational models of multisensory integration (Beck et al., 2008; Magnotti and Beauchamp, 2017).

There is a substantial body of evidence showing that posterior STS is a cortical hub for multisensory integration, responding to both auditory and visual stimuli including faces and voices, letters and voices and recordings and videos of objects (Calvert et al., 2000; Foxe et al., 2002; Beauchamp et al., 2004; van Atteveldt et al., 2004; Miller and D’Esposito, 2005; Reale et al., 2007). The finding that the adjacent cortex in posterior STG is also important for multisensory integration has several ramifications. In a transcranial magnetic stimulation (TMS) study, integration of auditory and visual speech (as indexed by the McGurk effect) was disrupted with TMS targeted at the posterior STS (Beauchamp et al., 2010). The present results suggest that posterior STG may also have played a role in the observed disruption, and raise the possibility that electrical brain stimulation of STG in ECoG patients can increase our understanding of multisensory speech perception as it has for visual perception (Murphey et al., 2008; Rangarajan and Parvizi, 2016).

While there have been numerous previous fMRI studies of noisy and clear audiovisual speech (e.g., Callan et al., 2003; Sekiyama et al., 2003; Bishop and Miller, 2009; Stevenson and James, 2009; Lee and Noppeney, 2011; McGettigan et al., 2012), none described a sharp boundary in the response patterns to clear and noisy speech within the STG. A likely explanation is that many of the previous studies use spatial filtering or blurring as a preprocessing step in their fMRI data analysis pipeline and reported only group average data, which introduces additional blurring due to inter-subject anatomical differences, especially for commonly-used volume-based templates. Combined, these two spatial blurring steps could easily eliminate sharp boundaries present in fMRI data. For instance, blurring eliminates the otherwise robust observation of functional specialization for different object categories in visual cortex (Tyler et al., 2003). Another possible explanation for the failure of previous studies to observe the boundary is the common practice of reporting responses only at the location of activation peaks, rather than examining the entire extent of the activation. Anterior and posterior STG form a continuous region of active cortex, with the strongest activation in anterior STG. Therefore, only reporting responses from a single peak STG location (which would almost certainly fall in anterior STG) would camouflage the very different pattern of activity in posterior STG.

### Implications for ECoG and fMRI

While the primary goal of our study was not a comparison of the two methodologies, there was good correspondence between the actual fMRI signal and the fMRI signal predicted from our measure of ECoG amplitude, the broadband high-gamma response in the window from 70 Hz to 110 Hz. This is consistent with mounting evidence that the high-frequency broadband signal in ECoG is a good match for the fMRI signal (reviewed in Ojemann et al., 2013). Other ECoG measures, such as the narrowband gamma response (30–80 Hz) or the narrowband alpha response, may characterize neuronal synchrony rather than level of neuronal activity, and hence are poorly correlated with the BOLD signal (Hermes et al., 2017).

A reassuring finding from the present study is that we observed similar patterns of responses between ECoG patients with epilepsy and healthy controls viewing the same stimuli and performing the same task. This provides data to partially mitigate persistent concerns that ECoG patients may have different brain organization than healthy controls, reducing the generalizability of the results of ECoG studies. A related concern is the small sample size typical of many ECoG studies. Our ECoG dataset compared anterior and posterior STG responses in five individual hemispheres. Our fMRI dataset more than doubled this sample size (to 12 hemispheres) and the continuous sampling of the fMRI voxel grid provided more statistical power to identify the location of the anterior-posterior border. Increasing the sample size would allow additional characterization of individual variability in the anterior-posterior border.

One minor discrepancy between the ECoG and fMRI results was a larger relative amplitude for the favored stimuli in ECoG. For instance, anterior STG showed a nearly three-fold difference in the response amplitude to clear vs. noisy audiovisual speech (300% vs. 110%). The difference in fMRI was in the same direction but much smaller (0.37% vs. 0.24%). We attribute this to the ability of ECoG electrodes to sample small populations of highly-selective neurons, while the BOLD fMRI response spatially sums over larger populations of neurons, mixing more and less selective signals. This same pattern has been observed in other studies comparing fMRI with ECoG. For instance, in a study of the fusiform face area, the BOLD signal evoked by faces was approximately double that evoked by non-face objects while the broadband high-gamma amplitude was triple or more for the same contrast (Parvizi et al., 2012).

## Author Contributions

MO and MSB designed and conducted the experiments, analyzed the data and wrote the manuscript. DY assisted with ECoG experiments.

## Conflict of Interest Statement

The authors declare that the research was conducted in the absence of any commercial or financial relationships that could be construed as a potential conflict of interest.

## Footnotes

^1^https://surfer.nmr.mgh.harvard.edu/optseq
